# Nutrition-associated health levels in cancer patients based on the ICF: an expanded study of 300 cases

**DOI:** 10.3389/fnut.2026.1720603

**Published:** 2026-05-13

**Authors:** Jinjin Chen, Shuqian Sun, Hua Shen, Jiangang Liu, Feng Lin

**Affiliations:** 1 Department of Cardiology, The Second Affiliated Hospital, School of Medicine, Zhejiang University, Hangzhou, China; 2 School of Rehabilitation Medicine, Nanjing Medical University, Nanjing, Jiangsu, China; 3 Department of Rehabilitation Medicine, Nanjing Lishui People’s Hospital, Nanjing, Jiangsu, China; 4 Department of Medical Oncology, Sir Run Run Hospital, Nanjing Medical University, Nanjing, Jiangsu, China; 5 Department of Medical Oncology, Nanjing Lishui People’s Hospital, Nanjing, Jiangsu, China; 6 Center of Rehabilitation Medicine, The First Affiliated Hospital of Nanjing Medical University, Nanjing, China; 7 Department of Rehabilitation Medicine, Sir Run Run Hospital, Nanjing Medical University, Nanjing, Jiangsu, China

**Keywords:** assessment, cancer, cancer rehabilitation, international classification of functioning, disability and health, nutrition

## Abstract

**Background:**

Early assessment of nutrition-associated health levels in patients with cancer—encompassing functioning, activities, and participation—is increasingly recognized as essential for nutrition assessment and cancer rehabilitation. Using a combined approach of item response theory (IRT) and graph modeling, our previous study developed a 32-item two-parameter logistic model (2PLM) for assessing the nutrition-associated health levels of cancer patients. By expanding the sample size and updating the methods, this study aimed to refine and extend the previous findings.

**Methods:**

This study collected International Classification of Functioning, Disability, and Health (ICF) data from 300 cancer patients via a maximum variation sampling strategy, including 200 newly recruited patients and 100 from our previous study. Person abilities (*θ*) and item parameters were calculated by constructing an IRT model, and the benefit index was estimated using a graph model.

**Results:**

The study constructed a 43-item three-parameter logistic model (3PLM) with high reliability (Cronbach’s *α* = 0.945, latent class reliability coefficient (LCRC) = 0.967). The estimated *θ* showed a significantly strong correlation with the Mini-Nutritional Assessment (MNA) (*p* < 0.001, *r_winsorized_* = 0.73), the Patient-Generated Subjective Global Assessment (PG SGA) (*p* < 0.001, *r_winsorized_* = −0.74), and the Nutrition Risk Screening 2002 (NRS 2002) (*p* < 0.001, *r_winsorized_* = −0.60), indicating high validity. In the graph model, 33 items formed a maximal connected subnetwork and 30 items exhibited a significant benefit index.

**Conclusion:**

The study established a 43-item 3PLM for assessing nutrition-associated health levels in cancer patients and estimated both item parameters and the benefit index. By incorporating a larger sample size and updated analytical methods, the study extended prior findings and supports the utility of this integrated approach for nutritional assessment and cancer rehabilitation.

## 1 Introduction

Malnutrition is a prevalent but often underrecognized condition among individuals with cancer, significantly compromising treatment tolerance, increasing complications, impairing bodily function, reducing quality of life, and adversely affecting overall survival (1–3). The European Society for Clinical Nutrition and Metabolism (ESPEN) has emphasized the need for early identification and monitoring of nutritional status in cancer patients to ensure comprehensive supportive care (4). Despite this, many commonly used nutritional assessment tools focus primarily on risk screening while overlooking nutrition-related health levels—particularly in the domains of functioning, activity, and participation—as described within the International Classification of Functioning, Disability, and Health (ICF) framework.

To address this gap, our previous study developed and validated a 32-item ICF-based two-parameter logistic model (2PLM), offering a novel approach for assessing the nutritional status of cancer patients (5). This tool demonstrated good psychometric properties and provided a novel approach for profiling nutrition-associated health levels among cancer patients. However, limitations, including a small sample size and the presence of a ceiling effect, may restrict the generalizability and robustness of the findings.

In addition, determining which item would benefit most from intervention remains a practical challenge in the field of rehabilitation. Identifying the different influences of symptoms in a dynamic functioning map may hold promise for increasing rehabilitation efficacy in clinical practice (6). Tailoring rehabilitation to specific functions is crucial for improving overall functional status. Using items as nodes and relationships between items as edges, a functioning map can be constructed. Perturbation analysis enables the simulation of function-specific interventions, which helps to estimate the possible benefits and to assess each function’s projected influence on the functioning map.

To extend our earlier findings based on 100 patients, we enrolled an additional 200 participants using the same inclusion criteria. By performing an advanced analysis that integrated item response theory (IRT) and graph modeling, the study aimed to refine and extend the previous results.

## 2 Materials and methods

### 2.1 Subjects and data collection

This study is a cross-sectional research study, and participants were recruited as inpatients from two tertiary hospitals in Nanjing using the maximum variation sampling strategy (7). This strategy aims to include participants with diverse characteristics, and the common patterns that emerge from a high degree of variation can reflect the shared characteristics of this population. The inclusion and exclusion criteria, as well as the data collection procedures, were consistent with those applied in the previous study (5). The inclusion criteria were as follows: (1) patients diagnosed with cancer; (2) those aged ≥ 18 years old; and (3) those who provided informed consent. The exclusion criteria were as follows: (1) patients with unstable conditions; (2) those with severe primary diseases, such as cardiovascular, liver, kidney, or brain diseases; and (3) those with cognitive dysfunction or an inability to understand and answer questions. As part of the maximum variation sampling strategy, we did not restrict cancer types or clinical stages. Ethical approval was obtained from the Ethical Committee of a Nanjing local tertiary hospital (No. 2018-SR017).

### 2.2 Statistical analysis

**Figure 1:**
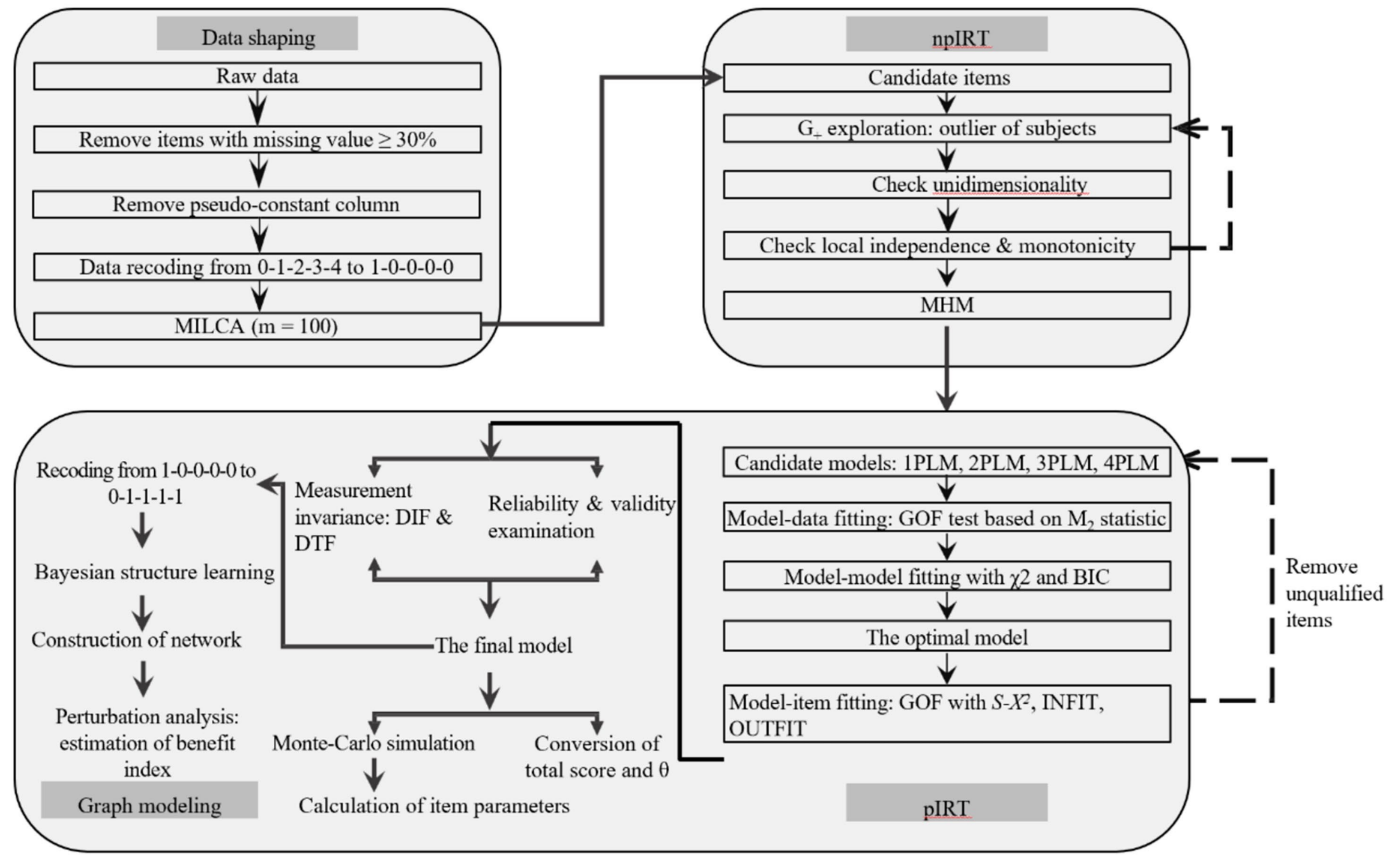
Flowchart of IRT modeling. NpIRT, non-parametric item response theory analysis; pIRT, parametric item response theory analysis; pseudo-constant column: the proportion of 0 or 1 ≤ 5%; MILCA, multiple imputation procedure based on latent class analysis; MHM, monotone homogeneity model; GOF, goodness of fitting; *χ*^2^, likelihood ratio test; BIC, Bayesian information criterion; *S-X*^2^, signed chi-squared test; INFIT, inlier-sensitive fit statistic; OUTFI, outlier-sensitive fit statistic; DIF, differential item functioning; DTF, differential test functioning. Items with missing values ≥ 30% were removed, and MILCA was applied to deal with missing values for items with missing values < 30%.

The IRT model was conducted in accordance with validated methodologies reported in previous research (8). After data preprocessing, four candidate IRT models were built, including the one-parameter logistic model (1PLM), two-parameter logistic model (2PLM), three-parameter logistic model (3PLM), and four-parameter logistic model (4PLM). The parameters include item difficulty (b), discrimination (a), guessing (g), and carelessness (u). An internal consistency reliability test, validity test, and measurement invariance test were performed on the best-fitting model. Additionally, a Monte Carlo simulation was used to generate 500 expected values of item difficulty. Figure 1 shows the detailed process of IRT modeling, including data preparation, IRT modeling, and model parameter calculation.

The dataset used for graph modeling was derived from the imputed data of items that were included in the optimal IRT model. In the graph modeling, data were recorded in order to analyze the risk correlations between items, where a higher score indicates greater impairment. Therefore, data were recoded from the original 1–0–0-0-0 format to 0–1–1-1-1. In other words, items with impairment were scored as 1, and items with no impairment were scored as 0. Bayesian structural learning (BSL) was used to calculate the posterior probabilities between items based on marginal pseudo-likelihood, with items as nodes, relevant relationships as edges, and interaction parameter (*β*) as edge weights. The graph with maximum posterior probability (MPP) was considered the highest probability of being the true structure. Edges with posterior probabilities greater than 0.5 were retained, and an Ising model was then constructed for further perturbation analysis (6). Perturbation was introduced by adjusting the node residuals in the Ising model by two standard deviations, with each residual reflecting the specific impairment of the node. The benefit index for each item was defined as the expected change in the overall system score resulting from the perturbation (deactivation) of that item. A higher benefit index indicated greater potential to improve the overall system, that is, the nutrition-associated health level. In this study, the benefit index was normalized.

### 2.3 Statistical methods and analysis tools

This study conducted data analysis using R software for Windows (version 4.2.0) (9). The correlation analysis and comparisons were performed using the ggstatsplot package (10). A *p-*value of < 0.05 was considered statistically significant during between-group comparisons. Enumeration data were described using frequency and analyzed using the chi-square test, with the effect size determined using Cramer’s V (11). Fisher’s exact test was performed when there was an expected frequency of less than 5. For measurement data, a normality test was initially performed using the Shapiro–Wilk test. Data with a normal distribution were described with their mean value and standard deviation and compared using the Welch t-test with an effect size of Cohen’s d (12). Data with a non-normal distribution were described with their median value and 95% confidence interval and analyzed using the Kruskal–Wallis test with an effect size of ε^2^ (13).

The study adopted the poLCA package (14) for data imputation, the Mokken (15) package for non-parametric IRT analysis, and the mirt package (16) for parametric IRT analysis and Monte Carlo simulation. The BDgraph package (17) and the bootnet package (18) were used to perform graph modeling.

## 3 Results

### 3.1 Demographic data

This study included 300 cancer patients in Nanjing and surrounding areas from September 2022 to June 2024 (7). Under the maximum variation sampling strategy, clinical heterogeneity between enrollment cohorts was an expected outcome designed to capture common patterns across a broad spectrum of patients; therefore, perfect baseline similarity was neither expected nor required. Baseline characteristics grouped by sex of the included participants are shown in Table 1. The study adopted rank epsilon squared (ε^2^) as the effect size for the Kruskal–Wallis test and Cramer’s V for Pearson’s chi-square test (11, 19). Although the two groups exhibited significant differences in age, body mass index (BMI), hemoglobin, albumin, C-reactive protein, and NRS 2002 score, the effect sizes were small (ε^2^ < 0.06). In clinical research, focusing on statistical significance alone can sometimes be misleading for clinical practice, particularly with a large sample size, where even small but clinically insignificant differences may be statistically significant. In our study, the observed effect sizes were consistently small and fell below the thresholds for a Minimal Clinically Important Difference (MCID) (20), defined as the smallest change in clinical outcome. Therefore, these differences, although statistically significant, were of negligible magnitude and are unlikely to be clinically significant.

**Table 1:** Baseline characteristics of participants.

Variable	All (*N* = 300)	Female (*N* = 129)	Male (*N* = 171)	Statistics (df)	*p*	ES	Method
Age group (<70/≥70 years)				*χ*^2^_(1)_ = 9.05	**<0.01**	**0.17**	Pearson *χ*^2^
Younger	186 (62.00%) 114 (38.00%)	93 (72.09%) 36 (27.91%)	93 (54.39%) 78 (45.61%)				
Older							
Operation				*χ*^2^_(1)_ = 0.11	0.74	0.02	Pearson *χ*^2^
No Yes	109 (36.33%) 191 (63.67%)	45 (34.88%) 84 (65.12%)	64 (37.43%) 107 (62.57%)				
Age (years)	67.00 (40.48, 83.00)	65.00 (35.20, 82.80)	69.00 (49.00, 82.50)	*χ*^2^_(1)_ = 16.14	**<0.01**	**0.05**	K-W
BMI (kg/m^2^)	21.97 (15.94, 29.57)	22.67 (16.42, 31.27)	21.48 (15.92, 27.62)	*χ*^2^_(1)_ = 6.82	**0.01**	**0.02**	K-W
Chemotherapy	5.00 (0.00, 31.10)	5.00 (0.00, 33.00)	4.00 (0.00, 23.50)	*χ*^2^_(1)_ = 1.77	0.18	0.01	K-W
Albumin (g/dL)	38.80 (27.45, 46.61)	39.60 (28.04, 47.14)	38.50 (25.95, 46.10)	*χ*^2^_(1)_ = 6.86	**0.01**	**0.02**	K-W
Hemoglobin (g/L)	111.50 (62.43, 143.00)	108.00 (71.40, 134.60)	114.00 (59.50, 144.75)	*χ*^2^_(1)_ = 7.63	**0.01**	**0.03**	K-W
CRP (mg/L)	3.06 (0.00, 119.19)	2.47 (0.00, 156.55)	3.77 (0.00, 94.45)	*χ*^2^_(1)_ = 4.57	**0.03**	**0.02**	K-W
MMSE	26.00 (15.00, 30.00)	25.00 (18.00, 30.00)	26.00 (14.23, 30.00)	*χ*^2^_(1)_ = 0.51	0.48	0.00	K-W
MNA	21.00 (8.24, 26.50)	22.00 (10.60, 26.40)	21.00 (7.12, 26.50)	*χ*^2^_(1)_ = 0.22	0.64	0.00	K-W
NRS2002	3.00 (1.00, 5.00)	2.00 (1.00, 5.00)	3.00 (1.00, 5.00)	*χ*^2^_(1)_ = 6.87	**0.01**	**0.02**	K-W
PG SGA	5.00 (1.00, 22.00)	5.00 (1.00, 18.00)	5.00 (1.00, 22.00)	*χ*^2^_(1)_ = 0.41	0.52	0.00	K-W
AND-ASPEN				*χ*^2^_(1)_ = 0.07	0.79	0.02	Pearson *χ*^2^
Malnutrition No malnutrition	141 (47.00%) 159 (53.00%)	59 (45.74%) 70 (54.26%)	59 (45.74%) 70 (54.26%)				

AND-ASPEN, malnutrition diagnostic criteria proposed by the Academy of Nutrition and Dietetics–American Society for Parenteral and Enteral Nutrition; BMI, body mass index; CRP, C-reactive protein; df, degree of freedom; ES, effect size; K-W, Kruskal–Wallis test; MMSE, Mini-Mental State Examination; MNA, Mini-Nutritional Assessment; NRS 2002, Nutritional Risk Screening 2002; PG SGA, Patient-Generated Subjective Global Assessment; Pearson *χ*^2^, Pearson’s chi-square test. Bold values indicate statistical significance (*p* < 0.05).

### 3.2 Construction of the IRT model

According to the sample size research of Straat et al. (21), the study used 0.42 as a scalability coefficient (H) for item selection. Through non-parametric IRT modeling, the study established a 48-item monotonicity homogeneity model (MHM) with a total H of 0.49, satisfying unidimensionality. The number of Guttman errors of subjects p022 and p118 was identified as extreme values, and they were removed. All H_ij_ of the 48 items were larger than 0, demonstrating local independence. Item characteristic curves (ICCs) exhibited a positively sloped, S-shaped form, indicating monotonicity.

**Figure 2:**
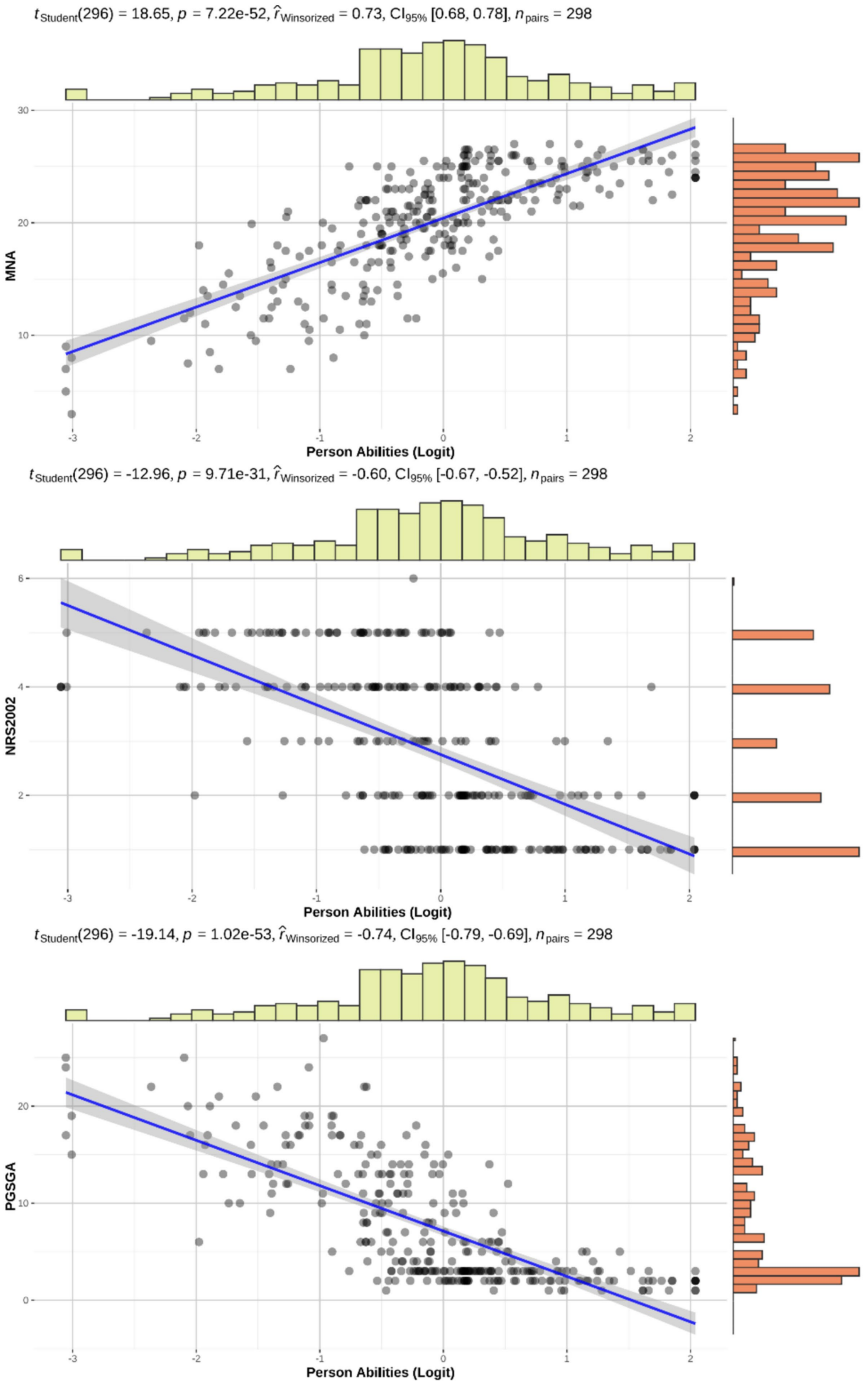
Correlation analysis between *θ* and MNA, NRS 2002, and PG SGA.

After a two-round iteration of parametric IRT analysis, a 43-item 3PLM was selected as the optimal model. As shown in Table 2, only 3PLM and 4PLM were well fitted. The likelihood ratio test indicated a significant difference between the two models with *p* = 3.75 × 10^−8^. We ultimately selected 3PLM as the optimal model because it yielded a lower Bayesian information criterion (BIC) (ΔBIC_3PLM-4PLM_ = −132.33). The selected 3PLM showed high reliability, with a Cronbach’s *α* of 0.945 and a latent class reliability coefficient (LCRC) of 0.967. Additionally, the estimated *θ* showed a strong, statistically significant correlation with the Mini-Nutritional Assessment (MNA) (*p* < 0.001, *r_winsorized_* = 0.73), the Patient-Generated Subjective Global Assessment (PG SGA) (*p* < 0.001, *r_winsorized_* = −0.74), and the Nutrition Risk Screening 2002 (NRS 2002) (*p* < 0.001, *r_winsorized_* = −0.60), as shown in Figure 2. This indicated that the 3PLM showed high validity.

**Table 2:** Item parameters of the 3PLM.

Item	Content	*a*	*b*	*g*	*b*_mc_	BI
d430※	Lifting and carrying objects	5.59	1.04	0	1.03	1*
d475※	Driving	1.99	0.98	0	0.96	0.51*
b730※	Muscle power functions	2.14	0.91	0	0.90	0.64*
b4552※	Fatigability	2.84	0.89	0	0.88	0.72*
b130※	Energy and drive functions	2.56	0.80	3.00 × 10^−4^	0.79	0.57*
d240※	Handling stress and other psychological demands	1.77	0.74	1.00 × 10^−4^	0.74	0.60*
b535※	Sensations associated with the digestive system	2.35	0.58	0.10	0.57	0.42*
d460※	Moving around in different locations	4.26	0.35	0	0.35	0.80*
***b250*** ※	Taste function	59.49	0.30	0.32	0.29	0.69*
***b1563*** ※	Gustatory perception	59.53	0.30	0.32	0.29	0.65*
b126※	Temperament and personality functions	2.21	0.16	0.04	0.16	0.46*
b144※	Memory functions	0.85	0.08	1.00 × 10^−4^	0.15	0.38*
d5701※	Managing diet and fitness	2.41	−0.03	0	−0.02	0.18*
d520※	Caring for body parts	5.74	−0.05	0	−0.04	0.61*
d510※	Washing oneself	6.91	−0.05	0	−0.04	0.60*
b164	Higher-level cognitive functions	1.59	−0.10	9.00 × 10^−4^	−0.07	0.24*
d470※	Using transportation	3.49	−0.13	0.01	−0.13	0.26*
b530※	Weight maintenance functions	2.71	−0.17	0.06	−0.17	0.04
***b280*** ※	Sensation of pain	1.25	−0.22	0.15	−0.22	0.35*
d435※	Moving objects with lower extremities	2.38	−0.27	0	−0.25	0.40*
d455	Moving around	4.23	−0.30	0	−0.29	0.37*
b510	Ingestion functions	3.02	−0.33	0.34	−0.32	0.15*
b545	Water, mineral, and electrolyte balance functions	0.99	−0.47	4.00 × 10^−4^	−0.38	0.28*
***b515***	Digestive functions	1.43	−0.44	0.12	−0.43	0.23*
d530	Toileting	5.65	−0.60	0.09	−0.59	0.15*
d450	Walking	3.51	−0.61	0.02	−0.60	0.27*
d420	Transferring oneself	4.44	−0.92	1.00 × 10^−4^	−0.90	0.07
***b520*※**	Assimilation functions	1.18	−1.00	8.00 × 10^−4^	−0.90	0.25*
b152※	Emotional functions	1.32	−1.04	1.00 × 10^−4^	−0.96	0.08
d410	Changing basic body position	4.68	−1.06	0	−1.05	0.13*
d465※	Moving around using equipment	1.78	−1.12	1.00 × 10^−4^	−1.07	0.09
d415	Maintaining a body position	4.41	−1.11	1.00 × 10^−4^	−1.09	0.06
b160	Thought functions	2.31	−1.17	1.00 × 10^−4^	−1.12	0.11*
d540※	Dressing	2.43	−1.21	2.00 × 10^−4^	−1.17	0.08
b140	Attention functions	1.63	−1.45	0.01	−1.37	0.09
***b4152***	Functions of veins	1.77	−1.61	6.00 × 10^−4^	−1.53	0.08
***d440***	Fine hand use	3.41	−1.66	1.00 × 10^−4^	−1.65	0.08
***d445***	Hand and arm use	3.76	−1.67	1.00 × 10^−4^	−1.65	0.06
b117	Intellectual functions	1.88	−2.03	2.00 × 10^−4^	−1.92	0.05
***b810***	Protective functions of the skin	1.18	−2.23	3.00 × 10^−4^	−2.00	0.21*
***b820***	Repair functions of the skin	1.10	−2.28	3.00 × 10^−4^	−2.01	0.21*
d550	Eating	1.60	−2.46	3.00 × 10^−4^	−2.30	0.06
d560	Drinking	1.97	−2.44	3.00 × 10^−4^	−2.32	−0.01

b, item difficulty; b_mc_, median value of item difficulty adjusted by Monte–Carlo simulation; a, discrimination; g, guessing; BI, benefit index. “*” indicates an item with a significant benefit index. The dashed line indicates θ of subject H. X.-Y., θ = −0.5038. Items labeled with ※ are the impaired items observed in H. X.-Y. Bold values indicate statistical significance (*p* < 0.05).

**Figure 3:**
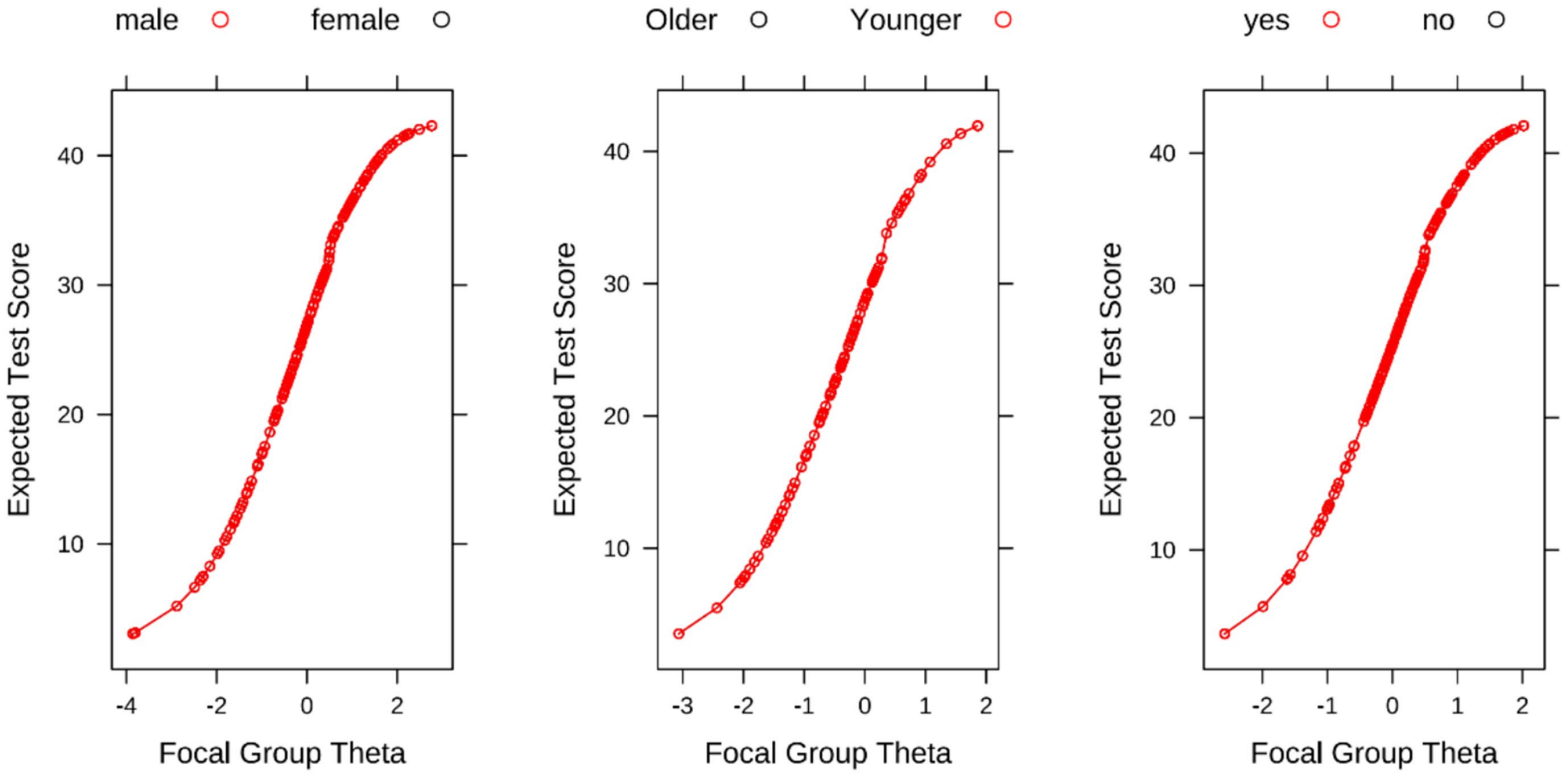
DTF analysis across sex, age, and tumor resection status.

A differential test functioning (DTF) analysis was performed across three variables: sex, age, and tumor resection status. The reference group was defined as female, age > 70 years, and non-surgical, while the focal group was defined as male, age ≤ 70 years, and surgical. The expected score curves of the 3PLM model overlapped across all subgroups (see Figure 3), indicating no evaluation bias and measurement invariance.

Using the Pearson correlation coefficient, *t*_Student_ represents the t-statistic (degrees of freedom in parentheses), *p* is the significance level, *r*_winsorized_ is the Winsorized correlation coefficient, CI_95%_ is the 95% confidence interval of the correlation, and *n*_pairs_ is the number of pairs included in the analysis. Top histogram: distribution of the latent trait; right histogram: score distributions of each scale.

Item characteristic curves (ICC) based on expected scores, grouped by sex, age, and surgical treatment. Red represents the focal group, and black represents the reference group. Group theta: person abilities of the focal group; older: older age group (age ≥ 75 years); younger: younger age group (age < 75 years); yes: underwent surgical treatment; no: did not undergo surgery.

**Figure 4:**
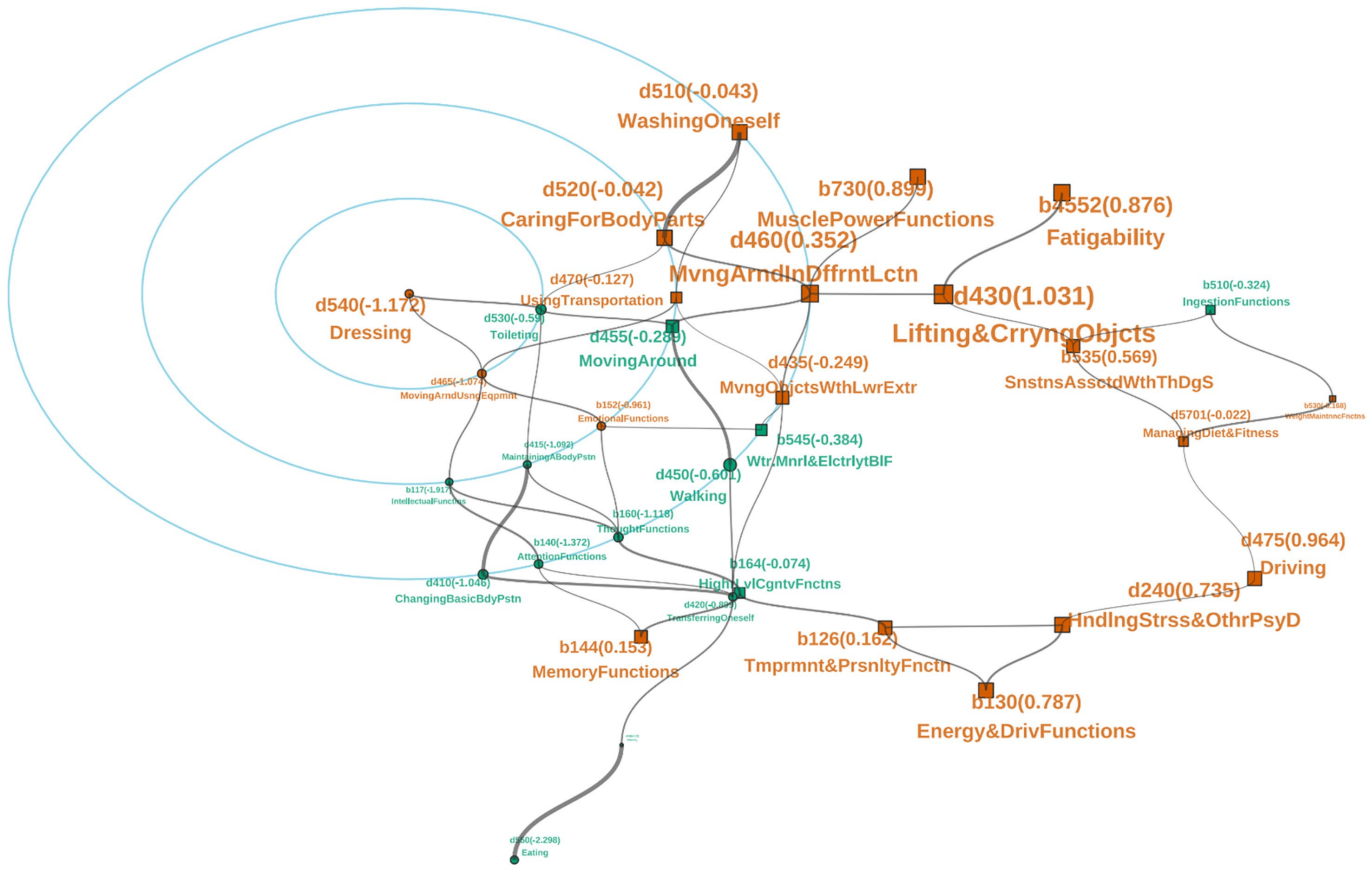
Visualization of the main component structure. Taking subject H.X.-Y., as an example, the layout of the network structure focused on the item with the lowest difficulty. Yellow denotes items with impairment, while green denotes items with no impairment. Items with difficulty exceeding θ are labeled as squares; otherwise, they are labeled as circles. The bigger the square or circle, the higher the benefit index of the item. The edge thickness indicates the strength of the interaction parameter in the Ising model; the thicker the edge, the larger the interaction strength. The number in the parentheses is the adjusted item difficulty.

Table 2 shows item parameters, including item difficulty, discrimination, and guessing parameters in the 3PLM. The study applied a Monte Carlo simulation to reduce sampling-induced error. The Kendall adjusted item difficulty correlation test was conducted to examine the association between adjusted difficulty and theoretical difficulty, yielding a strong positive correlation (*p* = 7.422 × 10^−21^, *τ* = 0.992). In the graph model constructed using these items, 33 items formed a maximal connected subnetwork, except for the 10 items in bold and italics in Table 2. Figure 4 is the visualization of the network of subject H. X.-Y. The layout of Figure 4 shows the item d540 (dressing), whose adjusted item difficulty was the smallest in the impaired items of H.X.-Y.

According to Austin (22) and MacGregor-Fors (23), the study used an 84% confidence interval (CI) to determine a *p-*value of < 0.05. Error bars represent 84% confidence intervals; non-overlapping 84% CIs indicate a statistically significant difference between group means at the *α* = 0.05 level. Thirty items exhibited a significant benefit index, with “*” labeled in Table 2. The benefit index and adjusted difficulty were significantly and strongly correlated, with a *p-*value of < 0.001 and *r* = 0.81.

## 4 Discussion

A comprehensive assessment of nutrition-associated health status (i.e., functioning, activities, and participation) is essential for the nutritional evaluation of cancer patients and for cancer rehabilitation. However, existing tools remain limited in fully capturing multidimensional health factors and guiding dynamic interventions. In this study, the combination of IRT and graph modeling not only deepened the understanding of the nutrition-associated health levels of cancer patients but also identified the key function nodes contributing to improvements in nutrition-associated health levels. This study provides an updated analysis comprising 300 individuals with cancer—including 200 newly recruited participants and 100 cases from our original cohort. By expanding the sample size and enhancing the analysis, we aimed to refine the previous results, particularly addressing their limitations in sample size and ceiling effects.

Grounded in the foundational assumptions of unidimensionality, local independence, monotonicity, and invariant item ordering, the IRT model was developed in this study. Previously, a 32-item 2PLM demonstrated high reliability and validity; in this study, a 43-item 3PLM was developed. Only b435 (immunological system functions) and b550 (thermoregulatory functions) were not represented in the revised scale, suggesting broad consistency and improved comprehensiveness. As shown in Table 3, the addition of items broadens the assessment of nutrition-associated health levels, enhancing the model’s ability to more precisely differentiate patients’ nutrition-associated health levels. Furthermore, the 3PLM also estimated a guessing parameter, providing additional information beyond the 2PLM.

**Table 3:** Comparisons of items of the 32-item 2PLM and the 43-item 3PLM.

Item	2PLM	3PLM	Content
b117		**+**	Intellectual functions
b126	**+**	**+**	Temperament and personality functions
b130	**+**	**+**	Energy and drive functions
b140		**+**	Attention functions
b144		**+**	Memory functions
b152	**+**	**+**	Emotional functions
b1563	**+**	**+**	Gustatory perception
b160		**+**	Thought functions
b164		**+**	Higher-level cognitive functions
b250	**+**	**+**	Taste function
b280	**+**	**+**	Sensation of pain
b4152		**+**	Functions of veins
b435	**+**		Moving objects with lower extremities
b4552	**+**	**+**	Fatigability
b510		**+**	Ingestion functions
b515	**+**	**+**	Digestive functions
b520	**+**	**+**	Assimilation functions
b530	**+**	**+**	Weight maintenance functions
b535	**+**	**+**	Sensations associated with the digestive system
b545	**+**	**+**	Water, mineral, and electrolyte balance functions
b550	**+**		Thermoregulatory functions
b730	**+**	**+**	Muscle power functions
b810	**+**	**+**	Protective functions of the skin
b820	**+**	**+**	Repair functions of the skin
d240	**+**	**+**	Handling stress and other psychological demands
d410	**+**	**+**	Changing basic body position
d415		**+**	Maintaining a body position
d420	**+**	**+**	Transferring oneself
d430	**+**	**+**	Lifting and carrying objects
d435	**+**	**+**	Moving objects with lower extremities
d440		**+**	Fine hand use
d445		**+**	Hand and arm use
d450	**+**	**+**	Walking
d455	**+**	**+**	Moving around
d460	**+**	**+**	Moving around in different locations
d465	**+**	**+**	Moving around using equipment
d470	**+**	**+**	Using transportation
d475		**+**	Driving
d510	**+**	**+**	Washing oneself
d520	**+**	**+**	Caring for body parts
d530	**+**	**+**	Toileting
d540	**+**	**+**	Dressing
d550		**+**	Eating
d560		**+**	Drinking
d5701	**+**	**+**	Managing diet and fitness

Items d510, d520, d430, d410, d420, d415, d460, and d455 showed high discrimination with low guessing, indicating a strong ability to differentiate among respondents. Therefore, the eight items might constitute a scale for self-assessment at home for patients with cancer. It was worth noting that two items exhibited unusually high discrimination (a ≈ 59) alongside the highest pseudo-guessing, which was a mathematical estimation artifact during the expectation–maximization (EM) algorithm with a 10^−5^ convergence threshold. Given the satisfactory goodness-of-fit, the items were retained in the final analysis. In addition, the item difficulty of d430, d475, b730, b4552, b130, d240, and b535 was high, indicating that they were challenging for patients. Functional impairments in these items might be associated with poorer nutrition-related health status and demonstrate limited improvements after interventions. Additionally, for items with a guessing value of 0 (e.g., d430), observed scores directly reflect the underlying trait level without inflation from guessing.

This study also provided an example of how the parameterized assessment tool can be applied to potentially help in clinical decision-making. The dashed line in Table 2 indicates *θ* of H. X.-Y., θ = −0.5038. By comparing θ and item difficulties, items below the dashed line could be categorized into the “functional competency zone” (b < θ), and items above the dashed line could be categorized into the “functional challenge zone” (b > θ). As shown in Table 2, items marked with “※” indicated impairments of H. X.-Y., and only five items with relatively small b-values in the functional challenge zone showed no impairment. However, four items within the functional competency zone exhibited impairments, in which the b-value of d540 was the smallest. Although d540 was the easiest item to improve, its benefit index was not significant, indicating that interventions targeting this item were unlikely to produce substantial improvements in overall health. The more difficult item, d435, within the main component network structure showed significant benefit, indicating a potential improvement in overall health. The layout of Figure 4 focused on item d540 (dressing), whose adjusted item difficulty was the smallest in H. X.-Y.’s impaired items. Item d470 was directly connected to item d435, with a significant benefit index and relatively small item difficulty. Therefore, d470 and d540 might be the appropriate targets for therapy.

In this study, the sample size was expanded, and the methodology and findings were further strengthened. However, there were still some limitations. First, a methodological limitation of this study is the potential for parameter estimation bias arising from the non-normal distribution of the latent trait in our sample. Since the expectation–maximization (EM) algorithm utilizes fixed quadrature based on a standard normal prior, this distributional mismatch can introduce conditional bias and slightly reduce measurement precision. Patients were recruited from Nanjing and the surrounding areas, which may limit the generalizability of our findings to broader populations. Future multi-site research using larger samples is recommended to further verify parameter stability. Second, the benefit index in this study is a model-derived potential estimate of system-level improvement rather than an observed outcome from an intervention trial. Further longitudinal intervention research is required as the essential next step to validate these findings. Third, although there were no completely matching scales for measuring activity and participation for comparison, some scales, such as the 36-item Short Form Survey (SF-36) and the 12-item Short Form Survey (SF-12), are still necessary to be applied and analyzed in further studies. Furthermore, due to the cross-sectional observational nature of this study and the lack of longitudinal follow-up, the predictive validity of the ICF-based model could not be assessed. Future prospective longitudinal studies are essential to evaluate the scale’s prognostic value regarding long-term clinical outcomes.

## 5 Conclusion

The study constructed a 43-item 3PLM with high reliability and validity, and these items encompass multiple aspects of nutrition-associated functioning, activities, and participation. Through the IRT model and graph model, item difficulty, discrimination, guessing, and the benefit index were estimated. These findings extend previous studies and provide a reference for individualized nutrition rehabilitation planning, offering new perspectives for nutrition assessment and cancer rehabilitation.

## Data Availability

The original contributions presented in the study are included in the article/supplementary material; further inquiries can be directed to the corresponding author.
